# Effects of Menadione on Survival, Feeding, and Tunneling Activity of the Formosan Subterranean Termite

**DOI:** 10.3390/insects12121109

**Published:** 2021-12-12

**Authors:** Kieu Ngo, Paula Castillo, Roger A. Laine, Qian Sun

**Affiliations:** 1 Department of Entomology, Louisiana State University Agricultural Center, Baton Rouge, LA 70803, USA; kngo4@lsu.edu (K.N.); pcastillo@agcenter.lsu.edu (P.C.); 2 Departments of Biological Sciences and Chemistry, Louisiana State University, Baton Rouge, LA 70803, USA

**Keywords:** termite management, vitamin K_3_, repellent termiticide, *Coptotermes formosanus*

## Abstract

**Simple Summary:**

Subterranean termites are wood-feeding insects that construct foraging tunnels in soil to search for cellulose-based food sources. The Formosan subterranean termite is an invasive species to the United States and one of the most destructive termites in the world. Management of these termites depends on understanding termite behavior and the use of termiticides that are effective, safe, and cost-efficient. Menadione, also known as vitamin K3, is safely used in animal feeds but toxic to some insects due to disruption of energy production by interference with mitochondrial respiration. In this study, we examined how menadione affected the survival, feeding, and tunneling activity of the Formosan subterranean termite. We found that when menadione was applied to food (paper) or tunneling substrate (sand), this compound caused mortality and reduced feeding activity in termites in a dose-dependent manner. When provided with both treated and untreated sand, termites were deterred from tunneling and feeding on the side treated with menadione. Overall, our findings demonstrated strong toxicity and repellency of menadione against the Formosan subterranean termite, suggesting this compound can be potentially used as a termite control agent.

**Abstract:**

The Formosan subterranean termite, *Coptotermes formosanus* Shiraki, is a highly destructive pest and a cosmopolitan invasive species. Sustainable termite management methods have been improving with the search for novel insecticides that are effective, safe, and cost efficient. Menadione, also known as vitamin K_3_, is a synthetic analogue and biosynthetic precursor of vitamin K with low mammalian toxicity. Menadione has shown insecticidal activity in several insects, presumably due to interference with mitochondrial oxidative phosphorylation. However, little is known about its effectiveness against termites. In this study, we evaluated the toxicity and repellency of menadione in *C. formosanus*. Our results showed that menadione affected the survival and feeding activity of termites both in filter paper and substrate (sand) treatments, and menadione influenced termite tunneling activity in treated sand. In a no-choice assay, ≥90% mortality after seven days and minimal or no food consumption were recorded when sand was treated with menadione at 6 to 600 ppm. In a two-choice assay with a combination of treated and untreated sand, termites were deterred by menadione at 6 to 600 ppm and exhibited low mortality (≤30%) over seven days, while tunneling activity was prevented with 60 to 600 ppm of menadione treatment. Overall, our study demonstrated dose-dependent toxicity and repellency of menadione in *C. formosanus*. The potential use of menadione as an alternative termite control agent is discussed.

## 1. Introduction

Subterranean termites (Blattodea: Rhinotermitidae) are wood-feeding insects that nest underground and construct extensive tunnels in soil to search for cellulose-based food sources. Pests in this group cause an estimated cost of USD 32 billion per year worldwide associated with treatment and repair of damaged structures [1]. The Formosan subterranean termite, *Coptotermes formosanus* Shiraki, is among the most destructive termites and recognized as one of the 100 worst invasive species in the world [2,3]. This species is native to eastern Asia, and invasive populations have been established in Hawaii and the southeastern United States [4,5]. In the U.S., the economic impact of *C. formosanus* was estimated to be USD 1 billion annually in 2005 [6], with a potentially increased cost in recent years due to the continued spread of invasive populations [7,8].

One of the primary treatment approaches for *C. formosanus* and other subterranean termites uses liquid termiticides to create an insecticide barrier in the soil around a structure [9]. The available products for soil treatment are divided into two categories, including repellents that are fast acting and non-repellents that are slow acting at low concentrations [9]. Repellent chemicals, mainly synthetic pyrethroids, are cost-efficient and prevent infestation by blocking termite access to the protected structure, but with reduced colony exposure and mortality due to the avoidance behavior of termites [10,11]. Non-repellent chemicals are toxic to exposed foraging termites and can be transferred to other colony members, which leads to high termite mortality and potential colony elimination [12,13,14,15]. Examples of non-repellents include fipronil, imidacloprid, indoxacarb, chlorfenapyr, and chlorantraniliprole [9], some of which are also toxic to beneficial organisms. Since the phasing out of cyclodiene insecticides, termite treatment methods have been improving in the direction of environmentally friendly and effective management incorporating the knowledge of termite behavior. However, adverse ecological impacts have been reported for some widely used liquid termiticides. For example, fipronil and neonicotinoids (e.g., imidacloprid) are toxic to aquatic invertebrates and fish, which poses potential risks to terrestrial animals, including humans, through consumption of aquatic products [16,17]. Fipronil is harmful to beneficial insects such as honey bees, and has been claimed as one of the main contributing factors to colony collapse disorder [18,19]. Extensive efforts have been made to develop sustainable termite management practices, including the application and improvement of termite baits based on chitin synthesis inhibitors [20,21,22] and the search for naturally derived organic compounds and their analogues [23,24].

Naphthoquinones are widespread organic compounds produced by plants [25]. Natural naphthoquinones, such as plumbagin and juglone, have been reported to exhibit insecticidal activities against a wide range of pests, including house flies [26], mosquitoes [27], red cotton bugs [28,29], whiteflies, and mites [30]. Menadione (2-methyl-1,4-naphthoquinone), also known as vitamin K_3_, is a naphthoquinone derivative and a synthetic analogue of vitamin K [31]. It functions as a vitamin precursor of K_2_ and utilizes alkylation to yield menaquinones. Although classified as a provitamin and allowed for use in animal feeds, its adverse effects prevent menadione from being used as a nutritional supplement in human food, and instead it may be used as an insecticide. Its unfavorable effects are caused by an unsubstituted position at C-3, which provides reactivity toward alkylating agents, and this depletes thiol levels and facilitates cytotoxicity. Therefore, these mechanisms result in vitamin K_3_ not being recommended as a dietary supplement for humans [31]. However, menadione can inflict oxidative stress and cause cell injury, and it was proposed to be a chemotherapeutic agent for cancer treatment [32,33,34,35]. Menadione is found to undergo reduction at complex I of the mitochondrial respiratory chain and diverts the electron flow to complex II [36]. Menadione as a benzoquinone can undergo one-electron reduction that produces a semiquinone radical, which reduces oxygen into a superoxide anion radical, while being oxidized back to quinone from the start [35]. This frequentative redox cycling production of excess intracellular reactive oxygen species may evoke oxidation of biological molecules in the mitochondrial matrix and cytosol, resulting in oxidative stress and apoptosis [35,37]. Although the actions of quinones vary with the specific compound and its concentration [25], in general, the interference with mitochondrial respiration is likely the mode of action for menadione as an insecticide. Osbrink and colleagues found that, by exposing termites of *C. formosanus* to treated filter paper, this compound caused 100% mortality at 0.5% (wt:wt) in three days and substantially reduced paper consumption [38]. However, behavioral responses of termites, such as tunneling and feeding, to menadione in experimental conditions simulating soil treatment were unknown, and menadione was not examined for repellency.

The overall goal of this study was to evaluate the effects of menadione on the survivorship, feeding, and tunneling behavior of termites in *C. formosanus*. Specifically, two feeding experiments with filter paper treatment were conducted, including a no-choice assay to determine the dose-specific influence of menadione on termite survivorship and feeding, and a two-choice assay with untreated and treated filter paper to test the repellent effect. In addition, two experiments simulating soil treatment were performed, which included a no-choice assay to evaluate the tunneling activity, food consumption, and survivorship of termites in sand treated with menadione, and a two-choice assay with untreated and treated sand to examine the repellent effect. To compare the effects of menadione with fipronil, a widely used termiticide, fipronil at a selected concentration was included in each experiment as a reference along with menadione treatment at different concentrations.

## 2. Materials and Methods

### 2.1. Termites

Colonies of *C. formosanus* were collected from the Brechtel Park in New Orleans, Louisiana, using underground milk crate traps filled with pine wood [39]. The collection sites for different colonies were separated by a minimal linear distance of 200 m. These colonies were maintained in the laboratory at 25 ± 1 °C in clear acrylic containers (38.48 × 45.72 × 22.86 cm^3^), provided with organic soil at the bottom and moistened pine wood logs as the food source. The relative humidity (RH) in each container was monitored weekly, and water was added to maintain 80–99% RH. All colonies were used for experiments within two months of collection. All experimental units described below were kept in an incubator at 27 ± 1 °C in complete darkness, and 80–99% RH was maintained.

### 2.2. No-Choice Filter Paper Feeding Assay

To examine the influence of menadione on termite mortality through feeding, four acetone solutions of menadione (≥98.0%, Sigma, St. Louis, MO, USA) at the concentrations of 0.1, 0.6, 1.0, and 6.0 µg/µL and one solution of fipronil (≥97.0%, Sigma, St. Louis, MO, USA) at the concentration of 0.6 µg/µL were prepared. The solvent (acetone) was tested as a negative control. Specifically, 100 µL of each solution (or solvent) was applied to a piece of filter paper (3.0 cm in diameter, Whatman grade 1, Cytiva, Marlborough, MA, USA). Upon evaporation of solvent, each paper was placed in a Petri dish (6.0 cm in diameter) and applied with 100 µL of distilled water, and then 30 termites (27 workers and three soldiers) were counted and introduced to the dish using feather weight forceps (BioQuip, Dominguez, CA, USA). Termite mortality was recorded daily over 14 days. The papers were oven dried at 60 °C for 30 min and weighed before and after the experiment to calculate food consumption. Each menadione and fipronil solution was tested in 12 replications (six each from two colonies), and the solvent control was replicated 10 times (five each from the same two colonies).

### 2.3. Two-Choice Filter Paper Feeding Assay

To test the repellent or attractant effect of menadione on termites, a negative control filter paper (2.0 cm in diameter) treated with solvent (acetone) was placed on one side of a Petri dish (6.0 cm in diameter). A separate 2.0 cm filter paper was placed on the other side and treated with one of the following: fipronil (0.6 µg/µL), one of the four concentrations of menadione (0.1, 0.6, 1.0, and 6.0 µg/µL), or another solvent control. Filter papers were oven dried as described above and weighed. A total of 50 µL of acetone or solution of chemicals to be tested was applied to each paper, and upon solvent evaporation, 50 µL of distilled water was added to each paper. Then, 30 termites (27 workers and three soldiers) were introduced to each Petri dish. Termite mortality was recorded daily over 14 days, and filter papers were oven dried and reweighed to determine food consumption. This experiment was replicated 12 times (six each from two colonies) for each tested chemical, and 10 times (five each from the same two colonies) for the solvent control.

### 2.4. No-Choice Sand Assay

**Figure 1:**
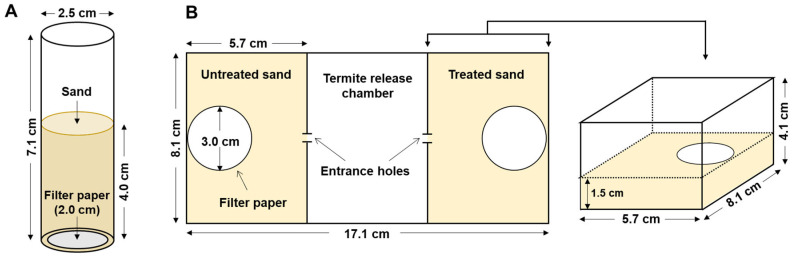
Schematic illustration of experimental setups. (**A**) Sand no-choice assay; (**B**) sand choice assay (top view of setup and side view of one compartment).

Plastic cylindrical containers (diameter: 2.5 cm; height: 7.1 cm; Qorpak, Clinton, PA, USA) filled with sand (Quikrete Premium Play Sand, New Orleans, LA, USA) were prepared (Figure 1A) to document mortality, sand penetration, and food consumption in termites. Specifically, 30 g of sand was treated with one of the following: solvent control (acetone), menadione at 1, 6, 10, 60, 100, and 600 ppm (wt [AI]:wt [sand]), and fipronil at 10 ppm. Each solution (or solvent) was mixed with sand in a 50 mL beaker, and the mixture was placed in a fume hood for 24 h in darkness to allow evaporation of solvent. Three pieces of pre-dried and weighed filter paper (2.0 cm in diameter) were placed at the bottom of each cylindrical container as a food source. The container was filled to 4.0 cm depth with treated sand, and 4 mL of distilled water was applied to moisten the sand. Fifty termites comprising 45 workers and five soldiers were introduced to each container. After seven days, the number of live termites was recorded, and the sand penetration depth was measured. Filter papers were oven dried and reweighed to calculate food consumption. Nine replications (three each from three colonies) were performed for this experiment.

### 2.5. Two-Choice Sand Assay

For testing the repellent or attractant effect of menadione through sand treatment, the experimental setup consisted of a rectangular plastic container (width: 17.1 cm; length: 8.1 cm; height: 4.1 cm) with three compartments (width: 5.7 cm; length: 8.1 cm; height: 4.1 cm each) (Pioneer Plastics, Dixon, KY, USA). Treated and untreated sand was contained on each side, and termites were released in the middle (Figure 1B). Entrance holes were drilled through the lower part of the dividers to allow movement of termites across compartments. For the treated side, 120 g of sand was treated with one of the following: solvent control (acetone), menadione at 1, 6, 10, 60, 100, and 600 ppm (wt [AI]:wt [sand]), and fipronil at 10 ppm. On the untreated side, sand of the same amount was mixed with only the solvent. The sand was mixed with each solution or solvent in a 150 mL beaker, dried under a fume hood for 24 h in darkness, and transferred to the left or right compartment of the container to a depth of 1.5 cm, with the addition of 16 mL of distilled water. One piece of pre-dried and weighed filter paper (3.0 cm in diameter) was placed on the top of sand away from the entrance hole. Fifty termites comprising 45 workers and five soldiers were introduced to the middle compartment of each container. After seven days, the number of termites present in the treated and untreated compartments was recorded, and overall mortality was documented for each container. Filter papers were oven dried and reweighed to calculate food consumption on each side. Nine replications (three each from three colonies) were performed for this experiment.

### 2.6. Statistical Analysis

All statistical analyses were performed utilizing the R software version 3.6.3 (The R Foundation, Vienna, Austria) [40]. Levene’s tests and Shapiro–Wilk tests were carried out to assess homogeneity of variances and normality of data distributions, respectively. Pairwise comparisons using the Wilcoxon signed-rank test were performed to determine significant differences between untreated and treated groups for paper consumption in the two-choice feeding and sand assays, as well as number of termites in the two-choice sand assay. All other statistical comparisons among groups were performed using generalized linear mixed models (GLMMs) with colony coded as a random factor and treatment coded as a fixed factor, followed by least square means, utilizing the lme4 package for R [41]. All original data were deposited in the Supplementary Materials (Table S1).

## 3. Results

### 3.1. No-Choice Filter Paper Feeding Assay

**Figure 2:**
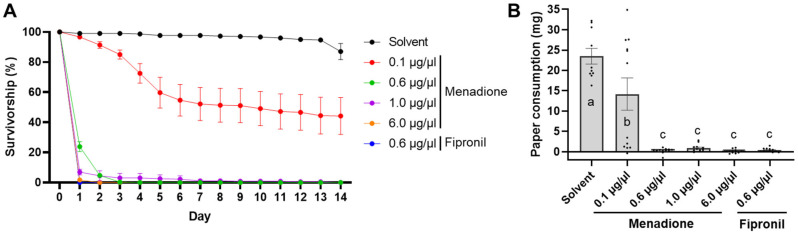
Influence of menadione on termite survival and feeding in a no-choice filter paper feeding assay. (**A**) Termite survivorship over time. Data are shown as mean ± standard error (SE). (**B**) paper consumption over 14 days. Bars represent mean ± SE, and dots show individual values. Groups denoted with the same letter are not significantly different (GLMM, *p* > 0.05; *n* = 10 for solvent, and *n* = 12 for each menadione or fipronil treatment).

In the filter paper treatment, survivorship of termites was affected by menadione in a dose-dependent manner (Table 1 and Figure 2A). No termites survived after one day of menadione treatment at 6.0 µg/µL, which was comparable to fipronil at 0.6 µg/µL. Less than 5% survivorship was observed at the menadione concentration of 0.6 and 1.0 µg/µL from day 3 to 14. When directly applied to filter paper, the LC50 of menadione was estimated to be 0.1 µg/µL for both day 7 and 14. Compared with the control, termites consumed significantly less filter paper at all tested menadione concentrations over 14 days, and no significant difference was detected among menadione concentrations of 0.6, 1.0, and 6.0 µg/µL and fipronil at 0.6 µg/µL (Figure 2B, GLMM, *df* = 62, *p* > 0.05).

### 3.2. Two-Choice Filter Paper Feeding Assay

**Figure 3:**
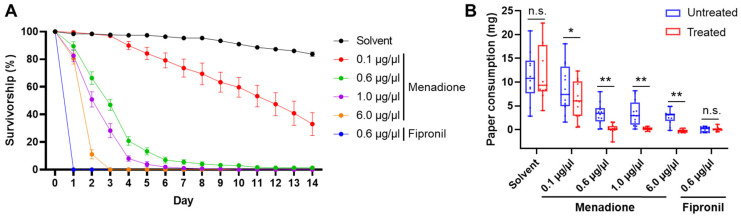
Repellent effect of menadione in a two-choice filter paper feeding assay. (**A**) Survivorship of termites over time. Data are shown as mean ± SE. (**B**) Consumption of untreated and treated filter paper over 14 days. Each box is bounded by the first and third quartiles, with the band indicating the median; whiskers represent the minimum and maximum; dots show individual values (Wilcoxon signed-rank test: n.s., not significant (*p* > 0.05); * *p* < 0.05; ** *p* < 0.01; *n* = 10 for solvent, and *n* = 12 for each menadione or fipronil treatment).

When exposed to both treated and untreated filter papers, more than 80% of the termites survived for one day at all tested menadione concentrations, and survivorship declined to 0% at 6.0 µg/µL in three days, while 0% survivorship was observed after one day with the fipronil treatment at 0.6 µg/µL (Table 2, Figure 3A). Less than 5% of the termites survived for 14 days under menadione treatment at 0.6 and 1.0 µg/µL. Termites consumed significantly less filter paper treated with menadione at all tested concentrations than the paired untreated filter paper, while no significant difference was found between the consumption of treated and untreated filter papers in the fipronil treatment and overall solvent control (Figure 3B, Wilcoxon signed-rank test, α = 0.05).

### 3.3. No-Choice Sand Assay

**Figure 4:**
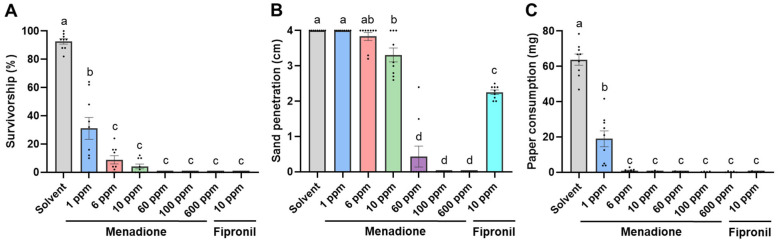
Influence of menadione on termite survival, tunneling and feeding activity after seven days in a no-choice sand assay. (**A**) Termite survivorship. (**B**) sand penetration depth. (**C**) filter paper consumption. Bars represent mean ± SE, and dots show individual values. Groups denoted with the same letter are not significantly different (GLMM, *p* > 0.05; *n* = 9 per group).

**Figure 5:**
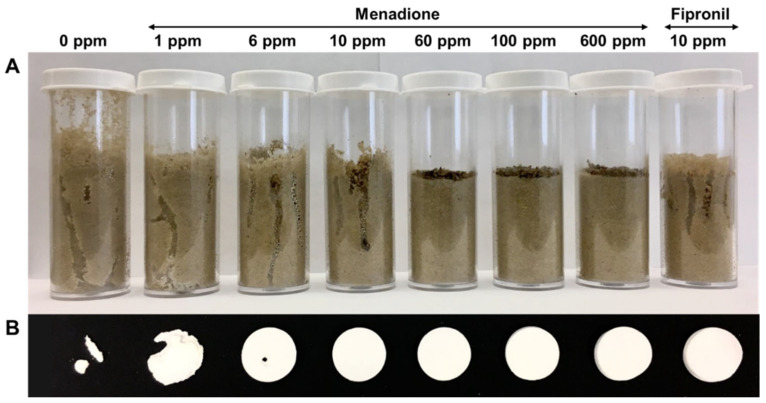
Representative images showing sand penetration (**A**) and filter paper consumption (**B**) after treatment for seven days in a no-choice sand assay.

A dose-dependent response was observed for termite survivorship when exposed to sand treated with menadione (Figure 4A). Compared with the control, treatments at 1 ppm or higher significantly reduced termite survivorship in seven days. The effects were not significantly different among the menadione treatments at 6 to 600 ppm and fipronil at 10 ppm (Figure 4A, GLMM, *df* = 62, α = 0.05). Over seven days, termites penetrated through the 4.0 cm depth of sand in control and menadione 1 ppm treatment, and the penetration depth was significantly reduced at 10 ppm. At 60, 100, and 600 ppm, insignificant penetration was observed (Figure 4B and Figure 5A, GLMM, *df* = 62, α = 0.05). In comparison, termites penetrated 2.26 ± 0.06 cm (mean ± SE) with fipronil-treated sand at 10 ppm (Figure 4B and Figure 5A). Filter paper consumption was significantly lower in all tested menadione concentrations (Figure 4C; GLMM, *df* = 62, *p* < 0.05). Paper damage was minimal at 6 ppm, with no damage observed at 10 to 600 ppm menadione or 10 ppm fipronil (Figure 5B).

### 3.4. Two-Choice Sand Assay

**Figure 6:**
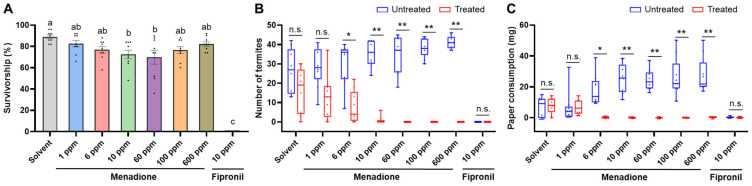
Influence of menadione on termite survival, distribution, and feeding activity over seven days in a two-choice sand assay. (**A**) Overall termite survivorship. Bars represent mean ± SE, and dots show individual values. Groups denoted with the same letter are not significantly different (GLMM, *p* > 0.05; *n* = 9 per group). (**B**) Number of live termites located in control and treated sand. (**C**) Filter paper consumption on the untreated and treated side. Each box is bounded by the first and third quartiles, with the band indicating the median; whiskers represent the minimum and maximum; dots show individual values (Wilcoxon signed-rank test: n.s., not significant (*p* > 0.05); * *p* < 0.05; ** *p* < 0.01; *n* = 9 per group).

**Figure 7:**
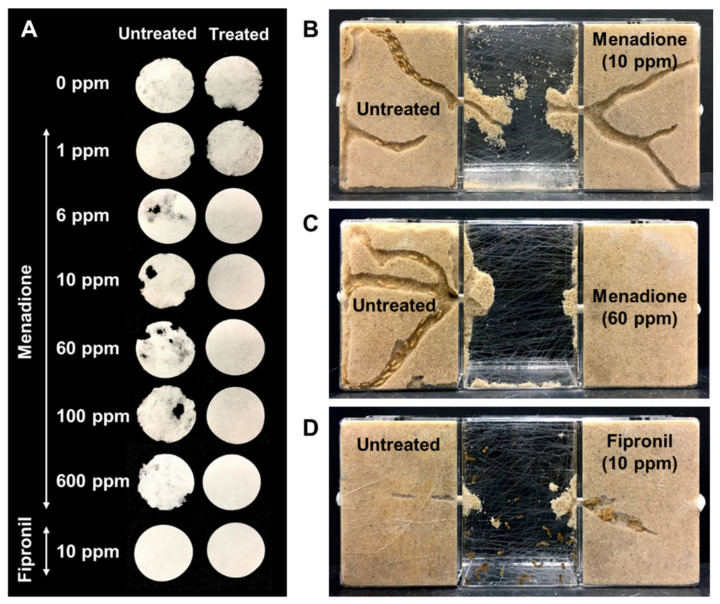
Representative images of consumed filter paper and foraging tunnels after seven days in a two-choice sand assay. (**A**) Paper consumption in the untreated and treated side. (**B**) tunnels are constructed in both untreated and treated sand with 10 ppm of menadione, with all live termites on the untreated side. (**C**) tunnels and live termites are shown in the untreated sand, but no tunnels are formed in sand treated with menadione at 60 ppm. (**D**) short tunnels are constructed on both sides, and dead termites are located within tunnels and in the middle compartment.

When termites were exposed to treated and untreated sand, the overall survivorship after seven days was not significantly affected by menadione at concentrations of 1, 6, 100, and 600 ppm, compared to the control (Figure 6A, GLMM, *df* = 62, *p* > 0.05). Menadione at 10 and 60 ppm significantly reduced termite survivorship (Figure 6A, GLMM, *df* = 62, *p* < 0.05), and the survivorship in these two groups was close to 70%; conversely, 0% survivorship was observed in all the replications for 10 ppm fipronil (Figure 6A). Menadione repelled termites and influenced filter paper consumption in a dose-dependent manner (Figure 6B,C). No significant difference in the number of live termites or paper consumption was detected between the untreated side and menadione treatment at 1 ppm. When sand was treated with menadione at 6 to 600 ppm, significantly fewer termites than in the untreated side were found and no paper consumption was observed. No live termites or paper consumption were found on either the untreated or treated side for fipronil at 10 ppm (Figure 6C and Figure 7A, Wilcoxon signed-rank test, *n* = 9 per group, α = 0.05). Foraging tunnels were observed on both the untreated side and the side treated with menadione at 10 ppm or lower (Figure 7B), while no tunnels were constructed in treatments with 60 ppm or higher (Figure 7C); termites briefly tunneled in both untreated and treated sand with fipronil at 10 ppm (Figure 7D).

## 4. Discussion

Our results showed concentration-dependent effects of menadione on the survival, feeding, and tunneling behavior of the Formosan subterranean termite, *C. formosanus*. Higher than 95% mortality was recorded after three days at 0.6 µg/µL of menadione (approximately 0.1% wt [AI]:wt [paper]) in a no-choice feeding assay. These findings demonstrated higher sensitivity of termites to menadione compared with a previous study, which used similar testing methods for *C. formosanus* and reported only 6.7% mortality after three days at 0.1% (wt:wt) and 100% mortality at 0.5% (wt:wt) of menadione [38]. Although menadione induced lower mortality than fipronil at the same tested concentration (Figure 2A), menadione was as effective as fipronil at 0.6 µg/µL in reducing filter paper consumption (Figure 2B). Additionally, in the no-choice sand assay, 10 ppm menadione was equally effective as fipronil at the same dose in reducing survivorship and consumption, and the same level of effect was observed as low as 6 ppm (Figure 4A,B). These findings suggest the potential use of menadione as a potent termiticide in protection of cellulose-containing materials.

Concentration-dependent repellency in termites has been reported for many synthetic pyrethroids, and the concentration threshold for preventing tunneling through soil varies across species [9,10,11]. With a choice assay, the avoidance concentration threshold of permethrin was 1 ppm for both *C. formosanus* and the eastern subterranean termite, *Reticulitermes flavipes* (Kollar), while *C. formosanus* was often less deterred than *R. flavipes* from tunneling through soil treated by other pyrethroids [11]. For example, deltamethrin and cyhalothrin were effective at 0.4 to 0.8 ppm to prevent soil penetration by *R. flavipes*, while 6 to 12 ppm concentration was necessary for *C. formosanus* [11]. These reported concentration thresholds were tested using glass tubes with moistened soil sandwiched between two sectors of agar and food, where termites were permitted to avoid treated soil. The conceptual design of these experiments was similar to our two-choice sand assay, where we found a strong repellency of menadione at 6 ppm against *C. formosanus* (Figure 6). Although termites could tunnel in sand treated at 10 ppm (Figure 7B), termites eventually moved away and caused negligible damage to treated filter paper (Figure 6B,C). In the no-choice assay where termites were constantly exposed to treated sand, tunneling activity was also seen at 10 ppm and reduced at 60 ppm (Figure 4B), which was higher than the mortality threshold of 6 ppm (Figure 4B), implying that menadione at concentrations between 6 and 60 ppm may provide temporary exposure and cause a low level of mortality in foraging termites. In the two-choice experiment, menadione at 10 and 60 ppm resulted in significant decreases in the survivorship than controls; however, overall survivorship remained high (>70%) between 1 to 600 ppm as termites were repelled (Figure 6A).

Menadione and other naphthoquinones have been shown to exhibit strong mitochondrial oxidative phosphorylation uncoupling and cytotoxic activity in mammalian cells [37,42]. Disruption of mitochondrial respiration may underlie the toxicity of menadione in *C. formosanus*, but the mechanism remains to be investigated in insect models. Given that energy production through mitochondrial oxidative phosphorylation is a ubiquitous process in the animal kingdom, menadione may have a broad-spectrum insecticidal activity similar to chlorfenapyr, a pyrrole insecticide [43,44]. Chlorfenapyr was effective as a slow-acting, non-repellent termiticide against multiple subterranean termite species, including *C. formosanus*, *C. gestroi* (Wasmann), *R. flavipes*, and *R. hesperus* Banks [45,46,47,48,49]. Our study indicates that menadione may be in the same mode of action category as chlorfenapyr, but with repellent activity. The insecticidal and repellent activities of menadione and other naphthoquinones in termites and other insect pests require additional studies.

Menadione, like the repellent pyrethroids, may provide an effective barrier against subterranean termites, but the reduced termite exposure and limited horizontal transfer within a colony make repellent compounds less desirable as soil treatment agents [9]. As termites are induced to change direction and forage through untreated soil, repellent termiticides require better application techniques and thorough treatment of all entry points compared with non-repellent termiticides [9,50]. However, due to its toxicity and deterrence in feeding, menadione can potentially be used for protection of interior wood and wood products. Some repellent termiticides, such as bifenthrin and permethrin, have been effectively used as wood preservatives under different settings [51,52,53,54]. The dose responses of different termite species, including subterranean and drywood termites, to various wood types treated with menadione warrant further investigation. In addition, the fast-acting property of menadione might allow its use in treatment of severe infestations, where the slow-acting chitin synthesis inhibitor baits may not be desirable.

In summary, our study revealed combined toxicity and repellency of menadione against *C. formosanus*. These effects, along with the low toxicity of menadione in mammals (about 0.5 g/kg orally in mice [55]), imply the potential use of menadione as a safe and cost-efficient termite control agent. To better understand how menadione and other naphthoquinones might be used for termite management, future research is necessary in several areas, including their possible effectiveness as wood preservatives, as well as toxicity and repellency to other termite species. As menadione is light sensitive and insoluble in water, another valuable future step is to develop formulations of this compound and its derivatives, and test their longevity under different environmental conditions.

## Figures and Tables

**Table 1:** Termite survivorship over time in a no-choice filter paper feeding assay. Within each column, groups denoted with the same letter in each row are not significantly different (GLMM, *p* > 0.05; *n* = 10 for solvent, and *n* = 12 for each menadione or fipronil treatment).

Concentration (µg/µL)		% Survivorship after Exposure (Mean ± SE)			
		Day 1	Day 3	Day 7	Day 14
Solvent		99.00 ± 0.51 a	99.00 ± 0.51 a	97.67 ± 0.87 a	86.00 ± 5.34 a
Menadione	0.1	96.67 ± 0.92 a	85.00 ± 3.06 b	52.22 ± 11.00 b	44.17 ± 12.26 b
	0.6	23.89 ± 3.23 b	0.28 ± 0.28 c	0 ± 0 c	0 ± 0 c
	1.0	6.94 ± 1.94 c	3.06 ± 3.06 c	1.11 ± 1.11 c	0 ± 0 c
	6.0	0 ± 0 cd	0 ± 0 c	0 ± 0 c	0 ± 0 c
Fipronil	0.6	0 ± 0 d	0 ± 0 c	0 ± 0 c	0 ± 0 c

**Table 2:** Termite survivorship over time in a two-choice filter paper feeding assay. Within each column, groups denoted with the same letter in each row are not significantly different (GLMM, *p* > 0.05; *n* = 10 for solvent, and *n* = 12 for each treatment).

Concentration (µg/µL)		% Survivorship after Exposure (Mean ± SE)			
		Day 1	Day 3	Day 7	Day 14
Solvent		98.33 ± 0.75 a	97.67 ± 0.71 a	95.33 ± 1.24 a	83.67 ± 1.61 a
Menadione	0.1	99.44 ± 0.37 a	96.94 ± 1.04 a	73.61 ± 6.52 b	33.06 ± 8.29 b
	0.6	89.44 ± 3.25 ab	49.94 ± 4.01 b	5.56 ± 1.71 c	1.39 ± 0.96 c
	1.0	82.50 ± 4.36 b	28.33 ± 5.15 c	1.11 ± 0.63 c	0 ± 0 c
	6.0	80.56 ± 4.02 b	0 ± 0 d	0 ± 0 c	0 ± 0 c
Fipronil	0.6	0 ± 0 c	0 ± 0 d	0 ± 0 c	0 ± 0 c

## Data Availability

The data presented in this study are available in the Supplementary Material.

## Supplementary Materials

The following are available online at https://www.mdpi.com/article/10.3390/insects12121109/s1: Table S1: Original data.
